# Retraction: Silencing of BAG3 inhibits the epithelial-mesenchymal transition in human cervical cancer

**DOI:** 10.18632/oncotarget.28708

**Published:** 2025-03-21

**Authors:** Fei Song, Geng Wang, Zhifang Ma, Yuebing Ma, Yingying Wang

**Affiliations:** ^1^Department of General Surgery, Shandong Provincial Third Hospital, Jinan, Shandong, China; ^2^Department of Emergency, Laiwu City People’s Hospital, Laiwu, Shandong, China; ^3^Department of Gynecologic Oncology, Shandong Cancer Hospital Affiliated to Shandong University, Shandong Academy of Medical Sciences, Jinan, Shandong, China

**This article has been retracted:** An Oncotarget investigation of this paper revealed overlapping invasion assay images in Figure 4A with those in Figure 4A of an earlier published paper from a different institution [1]. Additionally, Figure 4B contains manipulated western blot images previously used in Figures 4A and B of a second, unrelated publication [2]. We also found that the western blot of GAPDH expression in HeLa cells in Figure 4B is a duplicate of the GAPDH image from LoVo cells in Figure 3A of an unrelated 2013 paper [3]. Furthermore, the authors reused western blot Bag3 image from SiHa cells in Figure 2A in 2018 paper [4] to illustrate Bag3 expression in the tumor tissues.

Corresponding author Yingying Wang has since contacted Oncotarget to request a retraction of the paper, expressing the concern that the similarities between the figures in their study and previous studies raise doubts about the validity of their conclusions.

In light of these facts, the Editorial decision was made to retract the paper. We have received confirmation from all authors that they agree with this decision.

Original article: Oncotarget. 2017; 8:95392–95400. 95392-95400. https://doi.org/10.18632/oncotarget.20726
